# Reversal of axonal growth defects in an extraocular fibrosis model by engineering the kinesin–microtubule interface

**DOI:** 10.1038/ncomms10058

**Published:** 2016-01-18

**Authors:** Itsushi Minoura, Hiroko Takazaki, Rie Ayukawa, Chihiro Saruta, You Hachikubo, Seiichi Uchimura, Tomonobu Hida, Hiroyuki Kamiguchi, Tomomi Shimogori, Etsuko Muto

**Affiliations:** 1 Laboratory for Molecular Biophysics, Brain Science Institute, RIKEN 2-1 Hirosawa, Wako, Saitama 351-0198, Japan; 2 Laboratory for Molecular Mechanisms of Thalamus Development, Brain Science Institute, RIKEN 2-1 Hirosawa, Wako, Saitama 351-0198, Japan; 3 Laboratory for Neuronal Growth Mechanisms, Brain Science Institute, RIKEN 2-1 Hirosawa, Wako, Saitama 351-0198, Japan

## Abstract

Mutations in human β3-tubulin (*TUBB3*) cause an ocular motility disorder termed congenital fibrosis of the extraocular muscles type 3 (CFEOM3). In CFEOM3, the oculomotor nervous system develops abnormally due to impaired axon guidance and maintenance; however, the underlying mechanism linking *TUBB3* mutations to axonal growth defects remains unclear. Here, we investigate microtubule (MT)-based motility *in vitro* using MTs formed with recombinant TUBB3. We find that the disease-associated TUBB3 mutations R262H and R262A impair the motility and ATPase activity of the kinesin motor. Engineering a mutation in the L12 loop of kinesin surprisingly restores a normal level of motility and ATPase activity on MTs carrying the R262A mutation. Moreover, in a CFEOM3 mouse model expressing the same mutation, overexpressing the suppressor mutant kinesin restores axonal growth *in vivo*. Collectively, these findings establish the critical role of the TUBB3-R262 residue for mediating kinesin interaction, which in turn is required for normal axonal growth and brain development.

## Abstract (web summary)

How mutations in β3-tubulin cause axonal growth defects in congenital fibrosis of the extraocular muscles type 3 remains elusive. Minoura *et al*. develop a model system using recombinant human tubulin that demonstrates a link between tubulin mutation, impaired kinesin motility and axonal growth defects.

## Graphical abstract

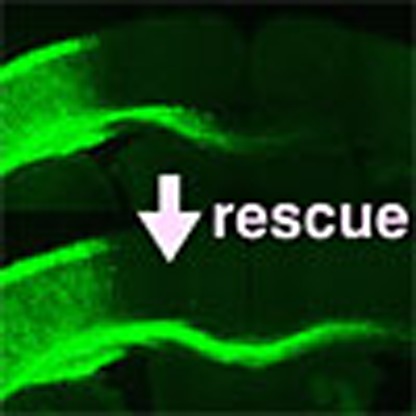

Microtubules (MTs), polymers of α/β-tubulin heterodimers, play critical roles in the developing nervous system^1^,^2^. Several distinct α- and β-tubulin isotypes are found in mammalian brain, and their tightly controlled spatiotemporal expression is essential for brain formation and function. Each tubulin isotype plays a specific role, and accordingly, mutations in tubulin genes are linked to neurological diseases with distinct phenotypes in an isotype-specific manner^3^,^4^,^5^.

Among the six β-tubulin isotypes expressed in the mammalian brain, class-III β-tubulin (TUBB3) is unique in its expression pattern in the nervous system. TUBB3 is primarily limited to neurons^6^, and confers a highly dynamic property to MTs compared with other β-tubulin isotypes^7^. Notably, the neuronal expression level of TUBB3 is highest during the period of axon outgrowth and is reduced in the adult central nervous system, suggesting that the dynamic property of TUBB3 is important for aspects of nervous system development^8^.

The uniqueness of TUBB3 is underscored by the fact that TUBB3's function cannot be replaced by other β-tubulin isotypes, not even by TUBB2, despite the 90% sequence identity between these two isotypes^9^. The class-III tubulin diverged from the common ancestor of class II, III and IV β-tubulins when vertebrates emerged; since then, this particular isotype has maintained 99% sequence identity across vertebrate species^8^ (Supplementary Fig. 1). Accordingly, the unique properties of TUBB3 critically depends on its exact sequence, and mutations in *TUBB3* in humans are associated with a spectrum of brain malformation and neurological disorders^9^,^10^,^11^. Specifically, eight heterozygous missense mutations in human *TUBB3* are reported to cause congenital fibrosis of the extraocular muscles type 3 (CFEOM3). Patients show hypoplasia of the oculomotor nerves and dysgenesis of the corpus callosum, anterior commissure and corticospinal tracts^11^. These defects point to an essential function for TUBB3 in the processes of axonal outgrowth and maintenance during development.

A subset of the *TUBB3* mutations causing CFEOM3 (R262C/H, E410K and D417H/N) has been studied in *Saccharomyces cerevisiae*, where the mutant β-tubulins were found to impair the MT interaction with Kip3p and Kip2p kinesins. Therefore, axonal growth defects in patients may likely result from compromised kinesin–MT interactions^11^. Nevertheless, although the involvement of TUBB3 acidic residues E410 and D417 in kinesin–MT interaction is supported by both *in vitro* and *in vivo* observations^12^,^13^, the involvement of basic residue R262 is uncertain. For example, a recent study in cultured mouse hippocampal neurons found no effect of the R262C mutation on KIF5B- or KIF21A-dependent transport^13^. The kinesin–MT interactions are primarily dependent on the interactions between the acidic residues in tubulin and the basic residues in kinesin^14^,^15^,^16^,^17^,^18^. Residue R262 has been neglected in these analyses because of its positive charge and its location in the H8-S7 loop, which is outside the C-terminal hairpin structures of α- and β-tubulin (H11–H11′–H12) where other critical acidic residues reside (Fig. 1). Because the R262 mutation is the most common mutation in patients with CFEOM3, delineating its role in kinesin motility is essential for understanding the disease mechanism.

A better understanding of whether and how R262 is involved in the kinesin interaction warrants a detailed examination of the effect of TUBB3 mutations on kinesin motility in a cell-free system. However, such an analysis has been hampered by a lack of methods to purify recombinant TUBB3 tubulin. To overcome this issue, we have recently developed a method to express and purify the heterodimer of human TUBA1B (α-tubulin) and TUBB3 tubulin using a baculovirus-insect cell expression system^19^. Here, using this new method, we demonstrate that TUBB3 R262H and R262A mutations inhibit the motility of kinesin *in vitro*. The reduced turnover rate of adenosine triphosphate (ATP) hydrolysis (*k*_cat_) indicates that the interaction via the R262 residue is critical for triggering ADP release from kinesin's nucleotide pocket. In agreement with this interpretation, the crystal structure of the kinesin–tubulin complex in its nucleotide-free state reveals that the R262 in tubulin binds with the D279 in the L12 loop of kinesin, which then connects to an element in the switch I/II subdomains^20^. With these biochemical and structural data, we sought a kinesin mutant that could bypass the effects of the TUBB3 R262A mutation and move on the mutant MT. We did indeed find one: in a proof-of-principle experiment, expressing this mutant kinesin in a mouse CFEOM3 disease model by *in utero* electroporation rescued axonal growth in the developing brain. Altogether, our findings establish that disruption of kinesin–MT interactions is a major cause for axonal growth defects in CFEOM3 patients.

## Results

### β-R262A mutation inhibits motility and ATPase activity

We used a baculovirus/insect cell expression system to express and purify the following three types of recombinant tubulin dimers composed of human α1-and β3-tubulin (TUBA1B and TUBB3, respectively): wild type (WT), R262C and R262H (Supplementary Fig. 2a)^19^. To compare our results with those from previously reported charged-to-alanine tubulin mutants^12^,^17^, the TUBB3 R262A mutant was also expressed. The WT, R262H and R262A tubulins were purified using His and FLAG tags and polymerized into MTs (Supplementary Fig. 2b), but the yield of R262C tubulin was too low for use in biochemistry experiments due to the low solubility of the protein.

**Figure 2:**
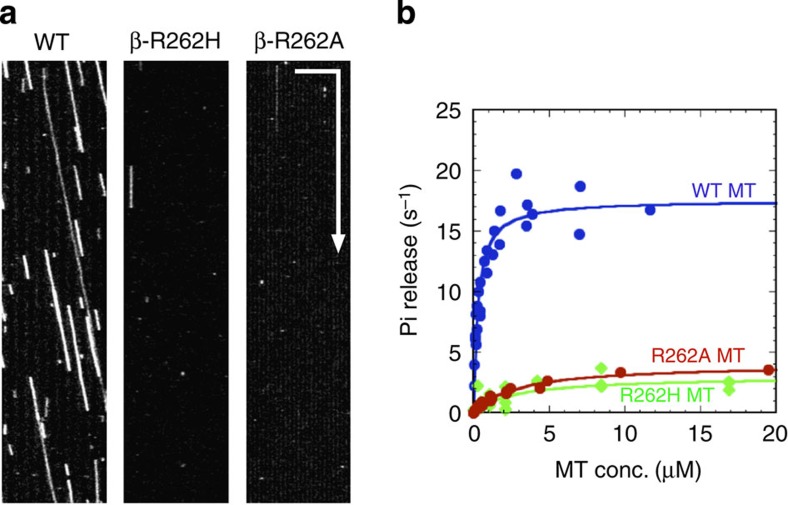
Effect of tubulin mutations β-R262H and β-R262A on single-molecule motility and MT-activated ATPase activity of the KIF5B motor. (**a**) Kymographs of kinesin motility. Horizontal bar, 5 μm; vertical arrow, 10 s. (**b**) The ATPase activity of KIF5B activated by WT (blue circles), R262H (green diamonds) or R262A MTs (red circles). All data obtained from four independent measurements for WT, three measurements for β-R262H and three measurements for β-R262A are plotted in the graph. Smooth curves were the best fit for the Michaelis–Menten equation with *k*_cat_ and *K*_0.5_MT values given in Table 1.

In the single-molecule motility assays using total internal reflection fluorescence microscopy, the two-headed KIF5B motor (human KIF5B construct HK432 at a concentration of 0.4 nM) did not bind to R262H or R262A MTs (Fig. 2a; Table 1), while it did move on the WT MTs at a velocity of 0.57±0.19 μm s^−1^ (mean±s.d., *N*=480). In the former cases, the kinesin scarcely bound to the mutant MTs even when the kinesin concentration was raised to 20 nM. Moreover, in MT-gliding assays using native KIF5, R262H and R262A MTs could not move on the kinesin-coated glass surface, while WT MTs moved at a velocity of 0.65±0.08 μm s^−1^ (mean±s.d., *N*=120). Therefore, the R262 residue in TUBB3 is critical for kinesin–MT interaction.

Measurement of MT-activated kinesin ATPase activity revealed that the R262H and R262A mutations reduced the ATP hydrolysis rate (*k*_cat_) to 17 and 23% of that of WT, respectively, and increased the value of apparent Michaelis–Menten constant (*K*_0.5_MT) 9.7 and 10 times of that of WT, respectively (Fig. 2b; Table 1). This result contrasts to our previous observation with the acidic critical residues in β-tubulin^17^. The charged-to-alanine mutations in acidic residues E410A and D417A, located in H12 of β-tubulin, have also been shown to impair kinesin motility. However, in the ATPase measurement, these mutations did not affect the value of *k*_cat_ (Supplementary Table 1). They only elevated the value of *K*_0.5_MT. The result indicates that basic residue R262 plays a distinct role, different from the role of other acidic residues in β-tubulin.

### A mutant kinesin rescues motility impaired by TUBB3 mutation

**Figure 3:**
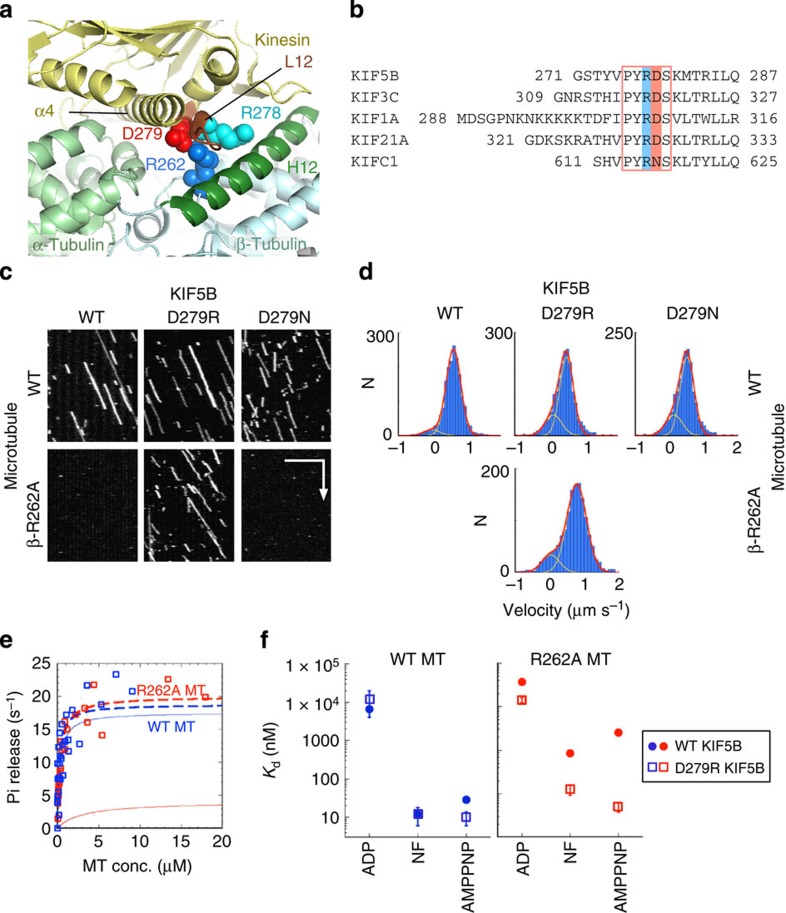
Motility rescue by D279R KIF5B. (**a**) Molecular structure of the kinesin–MT interface surrounding β-R262 of tubulin (PDB: 4LNU)^20^. A pair of charged residues, R278 (cyan spheres) and D279 (red spheres), in the kinesin L12 loop (brown) can be seen facing R262 in β-tubulin (blue spheres). (**b**) Alignment of L12 loop and the adjacent α5 helix sequences. The PYRDS motif (red box) is highly conserved in the kinesin families. (**c**) Kymographs of KIF5B motility on WT MTs and β-R262A MTs. Horizontal bar, 5 μm; vertical arrow, 5 s. Motility parameters are given in Table 1. (**d**) Histogram of the KIF5B velocities on WT and β-R262A MTs. (**e**) The ATPase activity of D279R KIF5B activated by WT MTs (□) or R262A MTs (□). All data obtained from four and three independent measurements (for WT and β-R262A, respectively) are plotted in the graph. Dashed curves represent the best fits to the Michaelis–Menten equation with *k*_cat_ and *K*_0.5_MT values given in Table 1. The ATPase activities of WT KIF5B (shown in Fig. 2b) are included as references (—, WT–MT; —, R262A MT). (**f**) Dissociation constants of the KIF5B–MT complex determined by a co-sedimentation assay. ‘NF' represents a nucleotide-free condition. Errors indicate errors in fitting the curve of the equilibrium binding data to the hyperbola (the raw data and exact number of *K*_d_ values are shown in Supplementary Table 2).

According to the crystal structure of the kinesin–tubulin complex^20^, β-R262 is a binding partner for D279 in the kinesin L12 loop in the nucleotide-free state (Fig. 3a). Adjacent to D279 in L12, there exists a highly conserved R278 (Fig. 3b), known to be most influential in determining the affinity of kinesin with the MT^14^. The substitution of R278 resulted in a 15-fold increase in the value of ATPase *K*_0.5_MT. Because the β-R262A mutation in tubulin shows a similar high impact on *K*_0.5_MT (Supplementary Table 1), we hypothesized that the role of β-R262 might be to guide the kinesin residue R278 to its binding partner (presumably D417 in the H12 helix of β-tubulin) by forming a salt bridge with the adjacent D279. According to this hypothesis, kinesin might move on the R262A MT if the residue R278 can bind to its partner residue(s) on an MT without being guided by β-R262.

To test this possibility, we attempted to engineer such a mutant kinesin that can move on an R262A MT by introducing the D279N and D279R mutations to the L12 loop of kinesin. Our expectation was that the reduction or reversal of the negative charge at D279 might reduce the electrostatic repulsion between D279 of kinesin and D417 and/or E421 of β-tubulin, allowing the adjacent R278 in kinesin to approach its binding partner(s).

In a single-molecule motility assay, both D279R KIF5B and D279N KIF5B moved on WT MTs at a velocity comparable to that of WT KIF5B (Fig. 3c,d, upper panel; Table 1). We found that D279R KIF5B moved on R262A MTs, but D279N KIF5B did not (Fig. 3c, lower panel). In an independent experiment, we confirmed that D279R KIF5B was not constitutively active; it could not move on β-E410A MTs, which was previously shown to be critical for kinesin motility^12^. Altogether, these results demonstrated that the mutation D279R, but not D279N, recovered the motility of KIF5B on R262A MTs. We also investigated whether the mutation D279R can recover the motility of KIF5B on R262H MTs, but the motility was not restored.

Along with motility, the D279R KIF5B mutation showed enhanced ATPase activity by R262A MTs (Fig. 3e; Table 1). The *K*_0.5_MT value was slightly higher than that of the WT–WT pairs.

Despite the recovered motility and ATPase activity in the paired mutant combinations of D279R KIF5B and R262A MT, their interaction differed from that of WT KIF5B–WT MTs in their motile and biochemical properties, as described below.

In a single-molecule motility assay, the binding frequency of D279R KIF5B to R262A MT was higher than that of the WT–WT combination (Table 1). Moreover, the movement of D279R KIF5B on the R262A MT was less smooth than the WT–WT combination. It stochastically passes between two modes, that is, a fast-moving mode and a sluggish mode, thus giving rise to the distribution of instantaneous velocities in two Gaussians (Fig. 3d). In the former mode, D279R KIF5B moved at a velocity slightly higher than that of the WT–WT combination (Table 1). In the latter, D279R KIF5B was either stalled at the same position or showed bidirectional drag along a R262A MT, thereby producing no net displacement. Such motility properties are unique to this paired mutant combination. Our previous analyses have shown that the charged-to-alanine mutations in the acidic residues of both α- and β-tubulin simply lowered the binding frequency and/or velocity of kinesin^17^. Regarding the binding affinity of the KIF5B–MT complex, a co-sedimentation assay using a minimal motor (a single-headed KIF5B construct, HK349) revealed that the affinity in the paired mutant combination (D279R KIF5B-β-R262A MT) was still lower than that of the WT–WT pair, showing a 2-, 10- and 2-fold increase in *K*_d_ values in the ADP, nucleotide-free and AMPPNP states, respectively, as compared with the WT–WT pair (Fig. 3f; Supplementary Fig. 3; Supplementary Table 2). Even though the affinity did not fully recover in the paired mutant combinations, the D279R mutation was highly effective in suppressing the R262A mutation effect, considering the large increase in *K*_d_ values brought about by the R262A mutation (∼6-, ∼70- and ∼90-fold increase in the ADP, nucleotide-free and AMPPNP states, respectively, as compared with the WT–WT pair).

### The mutant kinesin rescues axonal growth defects

**Figure 4:**
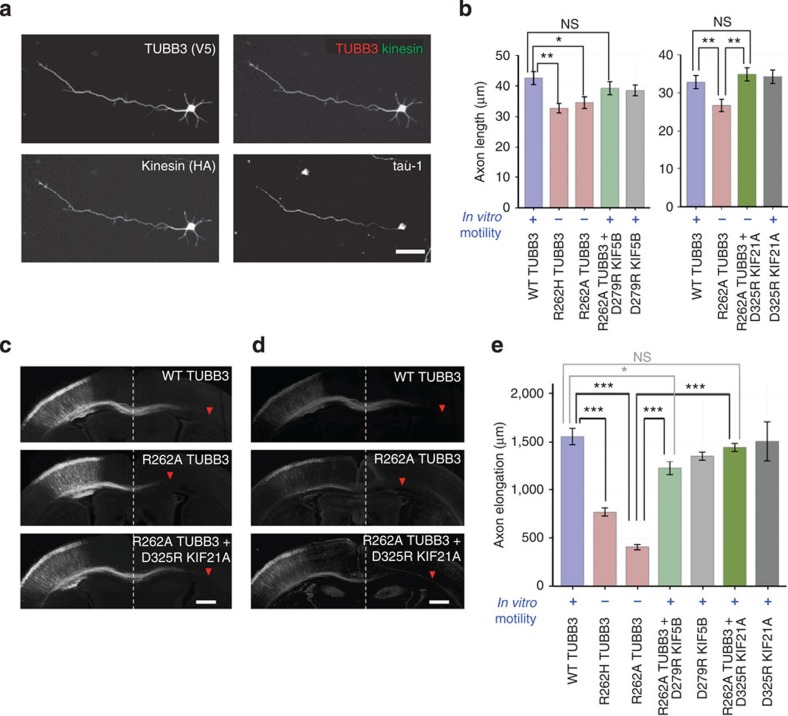
Rescue of axonal growth by kinesin mutants. (**a**) *In vitro* expression of mutant TUBB3 and KIF5B/KIF21A in mouse cortex neuron was detected by immunofluorescence via TUBB3-fused V5 tag or kinesin-fused HA tag. Cells were also stained for axonal marker, tau-1. Scale bar, 5 μm. (**b**) Effects of R262H and R262A TUBB3, D279R KIF5B and D325R KIF21A overexpressions on axon length. Lengths of 110–200 axons were measured. Error bars indicate s.e.m. *, ** and NS indicate *P*<0.05, *P*<0.01 and *P*≥0.05, respectively (ANOVA followed by *post hoc* pairwise Wilcoxon–Mann–Whitney tests). (**c**) Commissural axons in P3 mouse brain, transfected with (from above to below) WT *TUBB3*, R262A *TUBB3* alone and R262A *TUBB3* plus D325R *KIF21A*. Commissural axons were stained by anti-GFP antibody reacting with EYFP reporter. (**d**) Commissural axons were stained by antibody labelling V5 tag of recombinant TUBB3. (**c**,**d**) The broken line and red arrowheads indicate the midline and the tip of the axon, respectively. Scale bar, 500 μm. (**e**) Horizontal distances between the midline and the tip of the axon bundle, averaged from four to nine mouse brains (mean±s.e.m.). *, *** and NS indicate *P*<0.05, *P*<0.001 and *P*≥0.05, respectively (ANOVA followed by *post hoc* Tukey–Kramer tests). (**b**,**e**) Single-molecule motility of the equivalent combination, data for which are shown in Fig. 3 and Supplementary Table 3, is indicated in blue (+/−) below the graph. NS, not significant.

In light of the axonal growth defects in patients with CFEOM3 that result from mutations at R262, we next examined whether D279R kinesin rescued such defects in cultured neurons. Dissociated neurons from embryonic day 16.5 (E16.5) cerebral cortices were transfected with plasmids encoding WT, R262H or R262A TUBB3. After a 72- h culture, the lengths of the tau-1-positive axons in the cortical neurons were examined by immunocytochemistry (Fig. 4a). Compared with the control cells that were transfected with WT *TUBB3*, the transfection of R262H and R262A *TUBB3* perturbed the growth of axons, resulting in ∼20% reductions in axon length (mean±s.e.m., 42.5±2.2, 32.7±1.6 and 34.5±1.9 μm for the WT, R262H and R262A TUBB3, respectively; Fig. 4b; Supplementary Fig. 4), which was statistically significant (*P*=0.002 and *P*=0.035 for R262H and R262A, respectively, analysis of variance (ANOVA) with *post hoc* pairwise Wilcoxon–Mann–Whitney tests). In contrast, the cotransfection of D279R *KIF5B* with R262A *TUBB3* suppressed the reduction in axon length; the difference in axon length, as compared with the control cells, was insignificant (39.2±2.2 μm; *P*=0.30, ANOVA with *post hoc* pairwise Wilcoxon–Mann–Whitney tests). Thus, axonal growth was perturbed only in the conditions in which no motility was observed in the *in vitro* motility assay.

Because previous studies have implied a unique role of KIF21A in development of the nervous system^21^,^22^,^23^,^24^, in a separate set of experiments, we also tested the effect of cotransfection of D325R *KIF21A*, the mutation equivalent to D279R in *KIF5B*, with R262A *TUBB3* (Fig. 4b; Supplementary Fig. 4). While the transfection of R262A *TUBB3* caused significant reduction of axon length (mean±s.e.m., 32.8±1.7 for WT and 26.7±1.6 μm for R262A *TUBB3*; *P*=0.003, ANOVA with *post hoc* pairwise Wilcoxon–Mann–Whitney tests), the pair of mutants resulted in axon length comparable to that of control cells transfected with WT *TUBB3* (34.8±1.7 μm; *P*=0.63, ANOVA with *post hoc* pairwise Wilcoxon–Mann–Whitney tests). Again, the axonal growth perturbed by R262A TUBB3 was rescued by the suppressor mutation D325R in KIF21A. Expression of D325R KIF21A alone did not increase axonal length (34.2±1.8 μm; *P*=0.63, ANOVA with *post hoc* pairwise Wilcoxon–Mann–Whitney tests).

The rescue of axonal growth was even more prominent in an *in vivo* experiment in the developing mouse brain. In this experiment, a set of mutant kinesin and tubulin used in the above experiment were co-transfected with EYFP reporter into the cortical layer 2/3 commissure neurons at E15.5 with *in utero* electroporation^25^, and the elongation of commissural axons at postnatal day 3 (P3) was determined by immunolabelling for EYFP and TUBB3 (Fig. 4c,d). The elongation of TUBB3-positive axons was significantly reduced by the transfection of R262H or R262A *TUBB3* (mean±s.e.m., 1,558±85 for WT, 770±42 μm for R262H and 406±65 for R262A; *P*<0.001 for both of the *TUBB3* mutants, ANOVA with *post hoc* Tukey–Kramer tests, Fig. 4e). The cotransfection of D279R *KIF5B* and R262A *TUBB3* partially rescued the axonal growth. However, the axon length was still slightly shorter than that of the control cells transfected with WT *TUBB3* (1,226±69 μm; *P*=0.047, ANOVA with *post hoc* Tukey–Kramer tests). The cotransfection of D325R *KIF21A* and R262A *TUBB3* was more effective for the rescue and resulted in an axon length comparable to that of control cells (1,446±41 μm; *P*=0.93, ANOVA with *post hoc* Tukey–Kramer tests). We confirmed that the transfection of either D279R *KIF5B* or D325R *KIF21A* alone did not result in overextension of axons (Fig. 4e), indicating that neither of them were constitutively active. These *in vivo* observations, together with the results that were obtained with the cultured neurons, collectively indicate that the interaction between MTs containing TUBB3 and KIF21A/KIF5B is essential for normal axonal growth.

## Discussion

To date, there has been no evidence for a basic tubulin residue playing a critical role in kinesin-based motility^12^,^13^,^17^. However, a recent study in yeast showed that introducing the CFEOM3-associated mutation of the TUBB3 basic residue (R262C or R262H) to the yeast TUB2 gene blocked Kip3p accumulation at MT plus-ends^11^. Therefore, we aimed to investigate whether this basic residue mutation could inhibit the *in vitro* motility of kinesin when expressed in TUBB3. Our analyses revealed that the R262H or R262A mutations in TUBB3 impaired kinesin motility and the MT-activated ATPase of a two-headed KIF5B motor (Fig. 2; Table 1). Similar to an earlier study that reported a low yield of R262C tubulin in rabbit reticulocytes^11^, the poor yield of R262C TUBB3 prevented us from examining its effects on motility. However, the observation in yeast cells that both R262C and R262H mutations decreased Kip3p accumulation on the plus end of MTs indicated that R262C might impair kinesin motility if tested *in vitro*.

Having established the importance of the basic residue β-R262 in kinesin motility, our next questions were which residues of kinesin interact with β-R262 and how does this interaction contribute to the kinesin–MT interaction. A drastic reduction in *k*_cat_ by β-R262H or β-R262A mutations indicates that β-R262 might play a crucial role in mediating the structural signals required for ATPase activation. It contrasts with the role of the critical acidic residues in β-tubulin (β-E410 and β-D417) that increase the affinity of kinesin to MT (*K*_0.5_MT) without affecting *k*_cat_ (Supplementary Table 1)^17^. According to the crystal structure of the kinesin–tubulin complex and the model of the kinesin–MT complex based on cryo-EM images^20^,^26^,^27^, β-R262 is a binding partner for D279 in the kinesin L12 loop in the nucleotide-free and ATP-bound states (Fig. 3a). Upon MT binding, the rearrangement of salt bridges near this pair may contribute to the conformational change in kinesin, leading to ADP release from the nucleotide pocket^20^,^27^. The sequence of the L12 loop and the adjacent α5 helix is highly conserved in kinesin, indicating the importance of this region in its function (Fig. 3b).

Earlier work using alanine-scanning mutagenesis of kinesin showed that while the residue substitution at D279 reduced the value of ATPase *K*_0.5_MT to about half of that of the WT, the substitution in the adjacent R278 resulted in a more than 15-fold increase in this value (Supplementary Table 1)^14^. The latter 15-fold increase is outstanding, given that residue substitutions in all other kinesin sites brought at most only a fourfold increase in *K*_0.5_MT. This outstanding impact of R278A mutation in kinesin is comparable to the 10-fold increase in *K*_0.5_MT by the R262A tubulin mutation (Supplementary Table 1).

**Figure 5:**
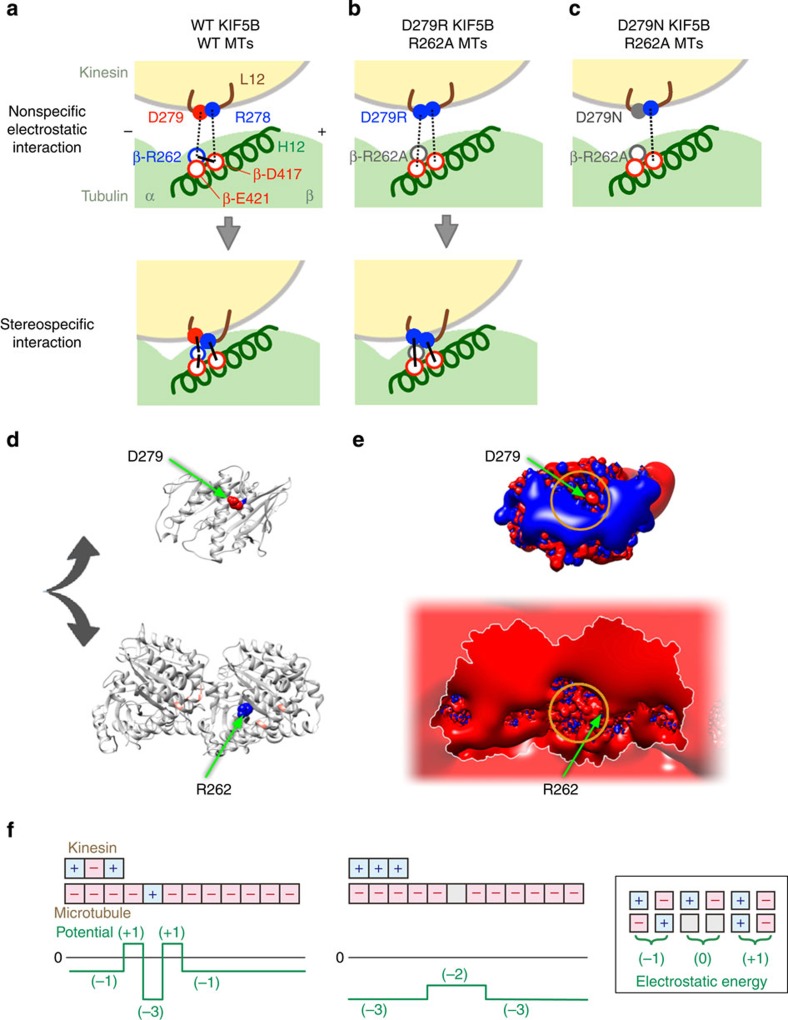
Schematic working model for the electrostatic latch. (**a**–**c**) Possible models for kinesin–MT interactions in the WT KIF5B–WT MT pair (**a**), D279R KIF5B-β-R262A MT pair (**b**) and D279N KIF5B-β-R262A MT pair (**c**). Red-, blue- and grey-filled circles represent basic, acidic and neutral residues in kinesin, respectively, whereas red, blue and grey unfilled circles represent basic, acidic and non-polar residues in tubulin, respectively. Dotted and thick lines indicate electrostatic attraction and the salt bridge, respectively. (**d**) Location of the residues D279 and R262 shown in the structure of KIF5B (top) and tubulin (bottom), based on the crystal structure of the KIF5B–tubulin complex in the nucleotide-free state^20^. (**e**) Three-dimensional isopotential contours for the KIF5B (top) and tubulin (bottom), calculated from the interface shown in **d**. The values of the contours are −2.5 *kT* *e*^−1^ (red) and 2.5 *kT* *e*^−1^ (blue) for the KIF5B and −26 *kT* *e*^−1^ (red) and 26 *kT* *e*^−1^ (blue) for tubulin, respectively, where *k* is the Boltzmann constant, *T* is the temperature and *e* is the magnitude of the electron charge. The arrows indicate the position of the residue D279 and β-R262. The yellow circles indicate the areas where the direction of the electrostatic field is locally reversed. (**f**) The schematic representation of the effect of the charge-reversed pair at the kinesin–MT interface on the electrostatic energy of the kinesin–MT interaction. For simplicity, the space has been one-dimensionalized and discretized. (Left) ‘Kinesin charges' (upper three boxes) electrostatically interact with ‘MT charges' (lower array of boxes), where the size of the box is roughly the Debye screening length^58^. The green line in the graph shows the electrostatic energy of the interaction between kinesin and MT, plotted as a function of the centre position of kinesin along the MT. The energy of each charge pair face-to-face is defined in the inset. In the WT KIF5B–WT MT pair (left), the energy landscape shows a distinct localized dip and peripheral banks, although the average binding energy is reduced as compared with that in the paired mutant D279R KIF5B-β-R262A MT (right).

Such similarly potent effects of kinesin R278A and tubulin R262A on *K*_0.5_MT values, and the close proximity of these two residues in three-dimensional space, suggest the possibility that β-R262 in tubulin may interact with D279 in the L12 loop of kinesin, and thereby mediate the encounter of adjacent R278 to its binding partner (presumably D417 in the H12 helix of β-tubulin) (Fig. 5a). In other words, because the MT is highly negatively charged, before (or concomitant with) binding with kinesin R278, the negative charge of D279 in the kinesin may need to be neutralized by electropositive β-R262 on the MT; otherwise, the D279 of kinesin and the D417 and/or E421 of β-tubulin will repel each other and prevent R278's access to the MT. This hypothesis is consistent with the local structural change at the binding interface, as shown in the models of kinesin–MT complexes^15^,^27^,^28^ and molecular dynamics simulations^29^.

This hypothesis predicts that kinesin might move on the R262A MT if the kinesin residue R278 can bind to its partner residue(s) on an MT without mediation by R262. Indeed, D279R KIF5B moved on a β-R262A MT (Fig. 3c,d; Table 1), indicating that a reversal of negative charge at D279 might have allowed the R278 to approach its binding partner without a charge neutralization of D279. In this mutant pairs, the lack of a D279-β-R262 (KIF5B–MT) salt bridge might have been compensated for by the electrostatic interaction of D279R-β-E421 (Fig. 5b). That D279N KIF5B did not move on R262A MTs is also consistent with our hypothesis; the electrostatic attraction between the putative binding sites is reliant on only a single pair of charged residues (Fig. 5c), which might be too weak for the kinesin–MT interaction to occur (see further discussion below).

In the D279R KIF5B-β-R262H MT mutant pairs, neither the motility nor the ATPase was rescued (Table 1). This result is not surprising considering the high histidine activity in molecular interactions^30^. Because histidine is capable of interacting with various amino acids, the mutation R262H might have modulated the MTs by hydrogen bonding(s), which cannot be compensated by D279R KIF5B^11^.

Our concluding question was what was the role of a pair with opposite charges in kinesin motility? In the paired mutant combination D279R KIF5B-β-R262A MT, its motile and biochemical properties differed from that of the WT–WT combinations in several aspects. A comparison between these two combinations provides us with insight into the unique role of basic residue R262 in motility.

Compared with the WT–WT pairs of kinesin–MT, in the mutant pairs, the interaction was less stable in all nucleotide conditions (that is, larger *K*_d_ in Fig. 3f), resulting in a shorter interaction duration in single-molecule motility and a slightly higher value of *K*_0.5_MT in the ATPase measurement (Table 1). These observations indicate that the binding free energy between D279R KIF5B and R262A MT is smaller than that in the WT–WT pair. In contrast to the two (or possibly three) salt bridges with identical polarities across the D279R KIF5B-β-R262A MT interface (Fig. 5b), the polarities of the two salt bridges across WT KIF5B–WT MT interface are opposite (Fig. 5a), which may make the latter complex more stable^31^.

On the other hand, the motility data showed that the binding frequency of D279R KIF5B to the R262A MT was two times higher than the binding frequency of WT KIF5B to WT MT (Table 1). Moreover, D279R KIF5B occasionally stalled or showed bidirectional drag along R262A MT (Fig. 3c,d). These results indicated the increased stability of the interaction between D279R KIF5B-β-R262A MT compared with that of the WT–WT pairs, which is contrary to the reduced stability of the interaction in solution (Fig. 3f).

These apparent discrepancies can be elucidated if we assume that the interaction between D279 and R262 had two opposite functions: it facilitates binding, but at the same time, it mediates detachment of the rear head from the MT during the processive run of two-headed kinesin undergoing ATP hydrolysis. We will explain this in the following paragraphs.

*Facilitated binding:* The interface of KIF5B for MT is dominated by positively charged residues, which produce a large area of enhanced positive electrostatic potential (Fig. 5d,e, upper panel)^18^,^32^. On the other hand, due to the high density of negatively charged residues, MT presents a streak of strong negative electrostatic potential that runs along the outer ridge of the MT protofilament (Fig. 5d,e, lower panel)^33^. These profiles of the electrostatic potentials allow KIF5B holding ADP to execute one-dimensional Brownian motion along the MT, while it is constrained in the negative potential of the MT^34^,^35^. With this background of the majority charges, the basic tubulin residue R262 and the acidic kinesin residue D279 are very few exceptions on the electropositive and electronegative surface of KIF5B and MT, respectively, reversing the potential landscape around their location (Fig. 5e, marked by yellow circles). Such local reversal in the potential landscape should help KIF5B locate its binding site during a diffusional search along MT, thus accelerating the subsequent transition to stereospecific binding (Fig. 5f, left)^36^. In the paired mutants D279R KIF5B-β-R262A MT, the lack of reversed charges, D279 and R262, might make a diffusional search of the binding site less efficient (Fig. 5f, right), causing prolonged diffusion in the weak-binding state (higher velocity compared with that of the WT–WT pairs) (Table 1; Fig. 3d, lower panel).

*Mediation of detachment:* For KIF5B bound on an MT, a small stagger in the relative position of D279 and R262, triggered by an external load, will result in each of these residues facing the like charges, thus allowing KIF5B to be ejected from the binding site (Fig. 5f, left). In the paired mutant D279R KIF5B-β-R262A MT, the lack of reversed charges may have eliminated the electrostatic repulsion, causing an ejection of the ADP-bound rear head from the MT difficult, and thus resulting in the stalling and/or dragging of kinesin (Fig. 5f, right).

In short, our model presumes that the reversed charge of D279 and R262 serves as an electrostatic latch, with its on and off being controlled by electrostatic attractions and repulsions. Although this model is currently speculative, it is consistent with the motility data (Table 1; Supplementary Fig. 5) and the results of the mutagenesis analysis of kinesin^14^. The validity of our model will be addressed in future studies by measuring the frequency and duration of stereospecific binding during diffusional scanning of the single-headed KIF5B along an MT under the ADP condition^37^,^38^.

Though our model is based on the results of an experiment using R262A MTs, it may explain the molecular mechanism underlying the impaired motility of the WT KIF5B on the disease-associated mutation R262H (Fig. 2). In this mutant, the reduction in the positive charge at residue R262 may result in MTs with an even surface potential, thus the electrostatic latch between R262 and D279 does not function.

Notably, a recent analysis of the critical residues in tubulin for dynein motility indicated that similar mechanisms may operate in this motor^39^. The basic residue α-R403 is essential for dynein to switch from diffusional movement to stereospecific binding, and its central importance is highlighted by the finding that a mutation in the equivalent residue in human TUBA3, α-R402H, disrupted dynein motility and caused lissencephaly^40^.

To compare the results of the *in vitro* analyses to the *in vivo* phenotypes of the disease, we recapitulated the axonal growth defect/rescue by overexpressing mutant TUBB3, KIF5B and KIF21A in cultured neurons and neurons in developing mouse brain (Fig. 4). In both experiments, the perturbation/recovery of axonal growth in neurons occurred in parallel with the perturbation/recovery of motility *in vitro*. While the overexpression of R262A TUBB3 inhibited axon growth, the coexpression of D279R KIF5B partially rescued the axonal growth perturbed by R262A, and the coexpression of D325R KIF21A led to a full recovery of axonal growth. The single-molecule motility assay confirmed that WT KIF21A showed no motility along R262A/R262H MTs, whereas D325R KIF21A moved on R262A MTs at a velocity of 0.16±0.45 μm s^−1^, which was comparable to that of WT KIF21A on WT MTs (0.18±0.32 μm s^−1^; Supplementary Table 3). These observations indicate that the interaction between the KIF motors and TUBB3 MTs was essential for axonal growth. Although R262A is a synthetic mutant, not reported in CFEOM3 patients, its similarity to disease-associated mutation R262H (that is, impaired motility and axonal growth defect) justifies its use as a model to investigate the mechanism underlying the pathogenesis. In agreement with our observation, a previous analysis of *in utero* electroporation demonstrated that the overexpression of TUBB3 with mutations at the acidic residues that are critical for kinesin motility (either E410K or D417H) perturbs axonal elongation in developing mouse brain^13^.

The extent of recovery in axon length was higher with D325R KIF21A than it was with D279R KIF5B. This may indicate distinct functions of these two motors during neuronal development. A recent immunohistochemical analysis of KIF21A knock-in mice indicated that KIF21A is essential for the molecular cascade controlling the steering of axonal growth^24^. Considering its condensed distribution in growth cones in a developing brain, KIF21A may bind to TUBB3-rich MTs at the growth cones and thus regulate the axon steering and elongation^23^,^24^,^41^. For KIF5B, an earlier study has shown that the suppression of KIF5 expression inhibits the polarization of hippocampal neurons^42^, indicating that this motor might be crucial for axon outgrowth. Accordingly, a recent analysis in *Drosophila* cultured neurons demonstrated that KIF5B-powered MT sliding is essential for the initial outgrowth of neurites^43^. The exact molecular mechanisms of how the interaction of TUBB3 with kinesin facilitates axon growth required further investigation.

In this study, the use of human TUBB3 tubulin allowed us to compare the results between *in vitro* and *in vivo* systems without having to deal with differences between the tubulin isotypes (for example, human TUBB3 versus yeast TUB2 tubulin)^11^. By extending this new approach towards the dissection of the pathogenic mechanisms in various neurological disorders, with each associated with a specific tubulin isotype^2^,^3^,^4^,^5^,^6^,^10^,^40^,^44^,^45^,^46^, we may be able to elucidate how different tubulin isotypes evolved to serve specific cellular functions in vertebrates.

## Methods

### Preparation of recombinant tubulins

Sequences of *Homo sapiens* α1-tubulin (TUBA1B; NP_006073) and β3-tubulin (TUBB3; NP_006077) were custom synthesized, with each clone fused with a glycine-based linker (GGSGG) and a peptide tag. For α1- and β3-tubulin, a His tag (HHHHHHHH) and a FLAG tag (DYKDDDDK) were used, respectively (Supplementary Fig. 2a)^19^. The codon usage of the sequences was optimized for expression in insect cells. To exclude variability due to acetylation, TUBA1B was made unacetylatable by residue substitution K40R and treated as WT. To increase expression levels, an L21 leader sequence^47^ was added to each of these sequences just before the start codon, and the inserts were cloned into the pFastBac Dual vector (Life Technologies, Carlsbad, CA, USA).

The recombinant tubulin vector was expressed in HighFive cells (Life Technologies) using the Bac-to-Bac System (Life Technologies). HighFive cells grown to a concentration of 2.0–2.5 × 10^6^ ml^−1^, were infected with the viruses and cultured for a further 48–72 h in suspension at 27 °C. Cells were collected (∼15 g wet weight from 1 litre of suspension culture) and lysed for 15 min in 90 ml of PMI buffer (0.1 M PIPES, 10 mM MgSO_4_, 2 mM EGTA and pH 6.8) supplemented with 0.5 M 3-(1-pyridinio)-1-propane sulfonate (NDSB201, Calbiochem, Merck KGaA, Darmstadt, Germany), 1% CHAPS, 5 mM dithiothreitol, 1 mM ATP, 1 mM GTP and protease inhibitors. This and the following steps were performed on ice or at 4 °C, unless otherwise stated. The lysate was centrifuged for 25 min at 200,000*g*. The resultant supernatant was supplemented with 10% (v/v) glycerol and adsorbed to 30 ml of DEAE Sepharose resin (DEAE Sepharose Fast Flow, GE Healthcare) for 60 min. After a wash with DEAE wash buffer (0.1 M PIPES, 10 mM MgSO_4_, 10% glycerol, 60 mM NaCl, 1 mM ATP, 1 mM GTP and protease inhibitors, pH 6.8), the retained protein was eluted with the same buffer containing 0.4 M NaCl, pH 7.0. This crude fraction (∼40 ml) was mixed with 4 ml of TALON resin (Clontech, Takara-Bio Inc., Ootsu, Japan) for 30 min. His-tagged tubulin was eluted with PMG buffer (0.1 M PIPES, 5 mM MgSO_4_, 10% glycerol, pH 7.0) supplemented with 0.3 M NaCl and 0.25 M imidazole. The eluate was diluted with an equal volume of PMI buffer supplemented with 10% glycerol, 1 mM GTP, 2 mM ATP and 0.04% NP-40, and mixed with 4 ml of anti-FLAG-tag antibody-conjugated resin (Sigma-Aldrich) for 1 h. FLAG-tagged tubulin was eluted with BRB80 buffer (80 mM PIPES, 2 mM MgCl_2_, 1 mM EGTA, pH 6.8) supplemented with 10% glycerol, 1 mM GTP and 0.2 mg ml^−1^ FLAG peptide (Sigma-Aldrich).

The purified tubulin was concentrated to 3–8 mg ml^−1^ with an Amicon Ultracel-30 K filter (Millipore, Merck KGaA), centrifuged to remove aggregation and then polymerized by adding 0.2–2 μM of taxol (Tocris) at 30 °C for 0.5–1.5 h. The polymerized material was supplemented with 2 mM ATP, 1 M NaCl and 20 μM Taxol, and then centrifuged for 12 min at 250,000*g*. The pellet was suspended in BRB buffer containing 1 mM GTP and 0.1 mM taxol at room temperature (RT). The MTs were stored at RT until use. Alternatively, tubulin dimer eluted from the FLAG affinity column was concentrated to >5 mg ml^−1^, frozen in liquid N_2_ and stored at −80 °C until use. Upon use, tubulin was thawed and further purified by a cycle of polymerization and depolymerization.

### Preparation of other proteins

To produce dimeric kinesin constructs HK432 and KIF21A-552, amino-acid residue 1–432 of KIF5B and residue 1–552 of KIF21A were each fused to a spacer (GGGGSGGGGS), a SNAP tag (New England Biolabs), a spacer (G) and a His6 tag, respectively. For the monomeric kinesin construct HK349, amino-acid residues 1–349 of KIF5B were fused to a spacer (GGGGSGGGGS), SNAP tag, a spacer residue (G) and a His6 tag (K349-SNAP-His). These constructs were expressed in *E. coli* then purified using immobilized metal-affinity chromatography (TALON, Clontech). The purified proteins were labelled with fluorescent dye BG-549 (SNAP-surface 549; New England Biolabs) at a dye-to-protein ratio of 2:1–5:1 for 60 min at RT, and separated from the free dye by gel filtration using a NAP-5 column (GE Healthcare) and by MT-affinity purification. The stoichiometry of the labelling was >0.9, as determined from the concentrations of both BG-549 and kinesin protein.

### Single-molecule motility assay

The surface of the glass slides (FF-001, Matsunami) were coated with biotin-functionalized polyethylene glycol (PEG) (BIO-PEG-SC molecular weight=3,400, Laysan Bio)^48^. A microscope chamber (18 mm × 7 mm × ∼30 μm) was constructed using a coverslip (#2918, Iwaki) and a PEG-coated glass slide. The MTs (either WT or R262A/H) were immobilized on the bottom of the chamber (=PEG-coated glass) via streptavidin and biotinylated nonmotile mutant kinesin, HK340-E236A-biotin^49^. A motility assay was conducted in × 2 MA buffer solution (× 2 motility assay buffer; 10 mM K-acetate, 20 mM PIPES-KOH, 4 mM MgSO_4_, 2 mM EGTA and 0.2 mM EDTA, pH 6.8) supplemented with 1 mM ATP, 1 mg ml^−1^ casein, 10 μM taxol, 1% 2-mercaptoethanol, and oxygen scavengers^50^ using BG-549-labelled HK432 at a concentration of 0.4 nM and a temperature of 25±1 °C. The motility was examined using total internal reflection fluorescence microscopy. A dark-field image of the unlabelled MTs and a fluorescent image of BG-549-labelled HK432 were simultaneously recorded using an EMCCD camera (ImagEM C9100-13, Hamamatsu Photonics Co.) over an observation period of <30 min (ref. 17).

### Image analysis of a single-molecule motility assay

The motion of each single BG-549-labelled kinesin was tracked using a tracking software (Mark2, kindly provided by Dr Ken'ya Furuta), and the data were processed and analysed using R statistical package (R Foundation for Statistical Computing) and in-house macro programs. The mean velocity was calculated by fitting the distribution of velocities (with a time interval of 0.3 s) to double-Gaussian curves. The mean run length and duration was determined by nonlinear least squares fitting of the data to the cumulative frequency distribution^51^, with the lower limit being set to 0.1 μm (run length) and 0.2 s (duration), respectively. The data were obtained by 3–4 independent experiments each containing >60 motor measurements.

### Multiple-motor motility assay

For an MT-gliding assay, a flow chamber prepared from a coverslip and a glass slide (9 mm × 9 mm × ∼80 μm) was loaded by first flowing casein solution (10 mg ml^−1^ of casein in 10 mM Tris, 0.1 M NaCl), waiting for 3 min and then sequentially chasing with the following solutions: (1) 12 μl of × 2 MA buffer solution; (2) 6 μl of × 2 MA buffer containing native kinesin (1–12 μg ml^−1^); and (3) 6 μl of the MT solution (10−50 μg ml^−1^ in × 2 MA buffer) supplemented with 1 mM ATP, 1 mg ml^−1^ casein and 10 μM taxol^12^. The chamber was sealed with nail enamel and the interaction of MTs with kinesin on the glass surface was observed under a dark-field microscope (BX50; Plan 40, numerical aperture=0.65, Olympus) at 25±1 °C. The images were projected onto an image-intensified charge-coupled device camera (C3077-70 and C8600-05, Hamamatsu Photonics) and stored in a digital video recorder (DR20, Sony). The kinesin density on the glass surface is estimated to be 200–2,400 μm^−2^ (refs 12, 52).

### Biochemical analysis of kinesin–MT interaction

MT-activated ATPase activity of WT and mutant HK432 was determined by the malachite green method at 25 °C (ref. 53). Equilibrium dissociation constant (*K*_d_) was measured by co-sedimentation of BG-549-labelled HK349 with MTs according to the standard method^54^ with some modification^34^. Assays were performed in × 2 MA buffer solution supplemented with 1 mg ml^−1^ casein, 40 μM taxol and either 1 mM ADP and 1 U ml^−1^ hexokinase (H500, Sigma), 1 U ml^−1^ apyrase (A-6132, Sigma) or 1 mM AMPPNP and 1 U ml^−1^ apyrase.

### Animals

Outbred ICR (CD-1) 10–12-week-old timed pregnant female mice were obtained from Japan SLC. Mid-day of the day of vaginal plug discovery was considered embryonic (E) day 0.5. This study was approved by the RIKEN Research Ethics Committee. Animals were handled under an animal use protocol approved by the committee.

### Primary culture and transfection of cerebral cortex

E16.5 embryonic cerebral cortices of the ICR mouse were treated with 0.25% Trypsin-EDTA for 5 min at 37 °C and dissociated into single cells by gentle trituration^55^. Cells were suspended in DMEM (Invitrogen) with 10% fetal bovine serum, then changed to Neurobasal medium (Invitrogen) supplemented with B27 (Invitrogen) and 2 mM L-glutamine (Sigma). Transfections were performed using an Amaxa mouse neuron nucleofector kit (Lonza, Basel, Switzerland), program O-005, according to the manufacturer's instructions. Upon completion of the program, cell suspension was combined with the pre-equilibrated Neurobasal medium and incubated in the CO_2_ incubator for 15 min for restoration, and then plated at a density of ∼60 cells per mm^2^ using Neuron Culture Medium (Sumitomo Bakelite Co., Tokyo, Japan) on coverslips coated with 1 mg ml^−1^ poly-L-lysine (Sigma) placed in film-bottom culture dishes (FD10300, Matsunami Co.). For transfections, plasmid DNA carrying *TUBB3*, *KIF21A* or *KIF5B* with a CAG promoter was used. For the TUBB3 constructs, a complementary DNA sequence used for overexpression in insect cells was used, in which the His tag was replaced with V5 tag for immunolabelling. For the KIF21A constructs, full-length mouse KIF21A (MGC:63452, MMM1013-202798465, OpenBioSystems) fused with a haemagglutinin (HA) tag was used. For the KIF5B constructs, a full-length human KIF5B (MGC:161557, NHS6278-211689013, OpenBioSystems) fused with a HA tag was used.

### Immunofluorescence cell staining and image acquisition

Cells were fixed in PBS with 4% paraformaldehyde and 4% sucrose at RT for 5 min followed by postfixation in −20 °C ethanol for 20 min. Fixed cells were washed three times with PBS (15 min each), permeabilized by 0.1% Triton X-100 in PBS for 10 min, washed three times with PBS (5 min each) and incubated in blocking buffer (3% BSA, 0.1% Triton X-100 in PBS) for 1 h at RT. The cells were then incubated with primary antibodies, either rabbit anti-V5 polyclonal antibody (2 μg ml^−1^, AB3792, Millipore) or rat anti-HA polyclonal antibody (2 μg ml^−1^, 11-867-423-001, Roche), and mouse anti-tau-1 monoclonal antibody (1/2 dilution, IHCR1015, Millipore) for 1.5 h. After three washes with PBS, cells were incubated with secondary antibodies, Alexa-Fluor 594-conjugated anti-rabbit immunoglobulin-G (IgG) (5 μg ml^−1^, A11012, Life Technologies), Alexa-Fluor 488-conjugated anti-rat IgG (5 μg ml^−1^, A11016, Life Technologies), and Alexa-Fluor 350 conjugated anti-mouse IgG (5 μg ml^−1^, A11045, Life Technologies) for 1 h at RT. After three washes with PBS, the cells were mounted with Fluoromount/Plus (Diagnostic Biosystems) and images were acquired with an IX-73 inverted microscope (Olympus) with a digital CMOS camera (ORCA-Flash 4.0, C11440, Hamamatsu Co.). The lengths of TUBB3 and/or KIF5B/KIF21A, and tau-1-positive neurites were measured by Simple Neurite Tracer^56^ for >100 transfected cells for each construct in each set of experiments. Statistical significance was determined by one-way ANOVA with *post hoc* pairwise Wilcoxon–Mann–Whitney tests for multiple comparisons using the R statistical package (R Foundation for Statistical Computing).

### *In utero* electroporation and immunohistochemistry

The plasmid DNA carrying EYFP with a CAG promoter was always co-injected with the plasmids carrying TUBB3-V5, KIF5B-HA and KIF21A-HA into the lateral ventricle and electroporated into the dorsal ventricular zone of the telencephalon at E15.5 (ref. 25). Transposon-mediated gene transfer was used for stable expression of tubulin and kinesin genes^57^. Brains were harvested at P3, sectioned in the coronal plane on a Leica sledge microtome at 20 μm and double-labelled for EYFP, and either the V5 tag of TUBB3 or the HA tag of KIF5B/KIF21A. EYFP was labelled with rat anti-GFP monoclonal antibody (2 μg ml^−1^, GF090R, Nacalai Tesque) and the secondary antibody conjugated to Alexa-Fluor 488 (10 μg ml^−1^, A11006, Life Technologies). V5 tag was labelled with rabbit anti-V5 polyclonal antibody (2 μg ml^−1^, AB3792, Millipore) and the secondary antibody conjugated to Alexa-Fluor 594 (10 μg ml^−1^, A11012, Life Technologies). The HA tag was labelled with rat anti-HA polyclonal antibody (2 μg ml^−1^, 11-867-423-001, Roche) and the secondary antibody conjugated to Alexa-Fluor 594 (10 μg ml^−1^, A11007, Life Technologies). To quantify the length of the axons, the horizontal distance from midline (Fig. 4c,d, broken lines) to the tip of the visible commissural axons (Fig. 4c,d, red arrowheads) was measured. Statistical significance was determined by one-way ANOVA with *post hoc* Tukey–Kramer tests for multiple comparisons.

## Additional information

**How to cite this article:** Minoura, I. *et al*. Reversal of axonal growth defects in an extraocular fibrosis model by engineering the kinesin–microtubule interface. *Nat. Commun.* 7:10058 doi: 10.1038/ncomms10058 (2016).

## Supplementary Material

Supplementary Information Supplementary Figures 1-5, Supplementary Tables 1-3 and Supplementary References

## Figures and Tables

**Figure 1:**
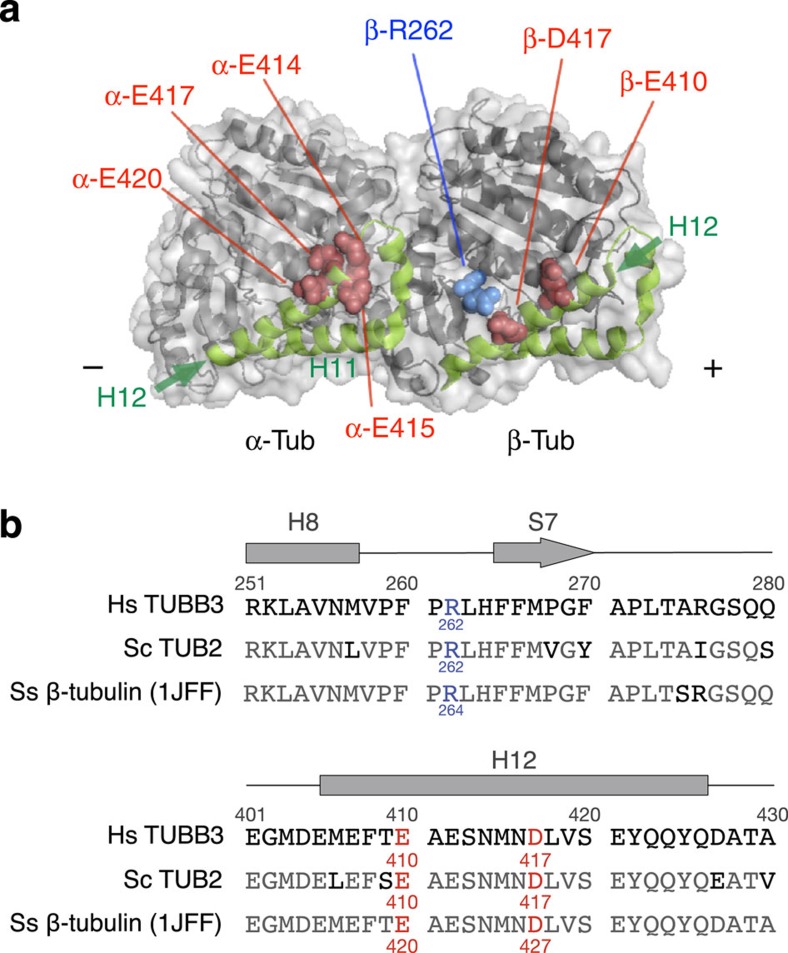
The position of residue β-R262 in the structure of tubulin dimer and in its amino-acid sequence. (**a**) Distribution of the residues involved in the interaction with kinesin on tubulin dimer (on face view of the MT; PDB: 1JFF). Alanine-scanning mutagenesis analysis of tubulin identified six acidic residues (red spheres) in the C-terminal hairpin structures (H11–H11′–H12) of α- and β-tubulin (green) as critical for the interaction with kinesin^17^. The basic residue β-R262 addressed in this paper (blue spheres) is located in the H8-S7 loop of β-tubulin. (**b**) Alignment of the sequences of human (Hs) TUBB3, *Saccharomyces cerevisiae* (Sc) TUB2, and pig (Ss) β-tubulin. For pig tubulin, sequence numbering used in the PDB 1JFF was adopted. The positions of the secondary structure elements are indicated, and the labelled residues correspond to those shown in **a** with the same colour scheme.

**Table 1:**
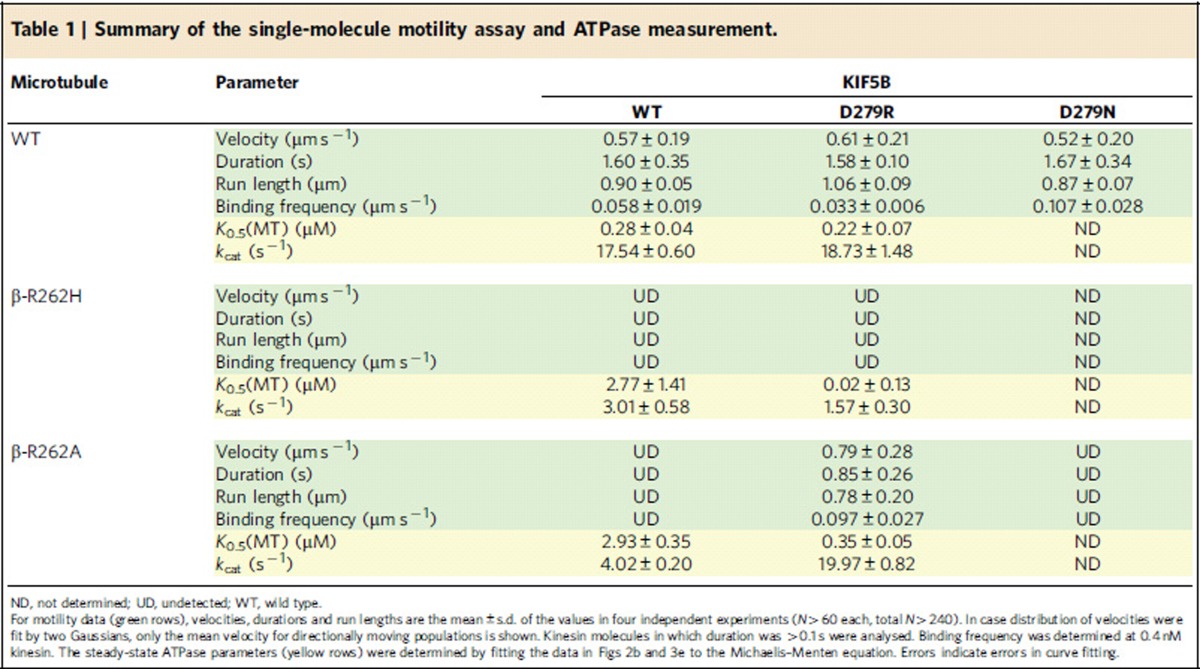
Summary of the single-molecule motility assay and ATPase measurement.
