# Supplementary material for: Reversal of axonal growth defects in an extraocular fibrosis model by engineering the kinesin–microtubule interface
Source: Nat Commun. 2016 Jan 18;7:10058. doi: 10.1038/ncomms10058 (PMC4735607; doi:10.1038/ncomms10058)
Supplement: Supplementary Information — Supplementary Figures 1-5, Supplementary Tables 1-3 and Supplementary References [file ncomms10058-s1.pdf]

```
                                        20                    40                    60                    80
hsTUBB3 (β3)      MREIVHIQAG QCGNQIGAKF WEVISDEHGI DPSGNYVGDS DLQLERISVY YNEASSHKYV PRAILVDLEP GTMDSVRSGA
mmTUBB3 (β3)      MREIVHIQAG QCGNQIGAKF WEVISDEHGI DPSGNYVGDS DLQLERISVY YNEASSHKYV PRAILVDLEP GTMDSVRSGA
ggTUB4B (β3)      MREIVHIQAG QCGNQIGAKF WEVISDEHGI DPSGNYVGDS DLQLERISVY YNEASSHKYV PRAILVDLEP GTMDSVRSGA
hsTUBB1 (β1)      MREIVHIQIG QCGNQIGAKF WEMIGEEHGI DLAGSDRGAS ALQLERISVY YNEAYGRKYV PRAVLVDLEP GTMDSIRSSK
mmTUBB1 (β1)      MREIVHIQIG QCGNQIGAKF WEVIGEEHGI DCAGSYCGTS ALQLERISVY YNEAYGKKYV PRAVLVDLEP GTMDSIRSSR
ggTUB6B (β1)      MREIVHIQAG QCGNQIGAKF WEVISDEHGI DPTGSYHGDS DLQLERINVY YNEAAGNKYV PRAILVDLEP GTMDSVRSGP
hsTUBB2B (β2B)    MREIVHIQAG QCGNQIGAKF WEVISDEHGI DPTGSYHGDS DLQLERINVY YNEATGNKYV PRAILVDLEP GTMDSVRSGP
mmTUBB2B (β2B)    MREIVHIQAG QCGNQIGAKF WEVISDEHGI DPTGSYHGDS DLQLERINVY YNEATGNKYV PRAILVDLEP GTMDSVRSGP
ggTUBB1 (β2B)     MREIVHLQAG QCGNQIGAKF WEVISDEHGI DPTGTYHGDS DLQLERINVY YNEATGGKYV PRAVLVDLEP GTMDSVRSGP
hsTUBB4 (β4A)     MREIVHLQAG QCGNQIGAKF WEVISDEHGI DPTGTYHGDS DLQLERINVY YNEATGGNYV PRAVLVDLEP GTMDSVRSGP
scTUB2 (β)        MREIIHISTG QCGNQIGAAF WETICGEHGL DFNGTYHGHD DIQKERLNVY FNEASSGKWV PRSINVDLEP GTIDAVRNSA

                                       100                   120                   140                   160
hsTUBB3 (β3)      FGHLFRPDNF IFGQSGAGNN WAKGHYTEGA ELVDSVLDVV RKECENCDCL QGFQLTHSLG GGTGSGMGTL LISKVREEYP
mmTUBB3 (β3)      FGHLFRPDNF IFGQSGAGNN WAKGHYTEGA ELVDSVLDVV RKECENCDCL QGFQLTHSLG GGTGSGMGTL LISKVREEYP
ggTUB4B (β3)      FGHLFRPDNF IFGQSGAGNN WAKGHYTEGA ELVDSVLDVV RKECENCDCL QGFQLTHSLG GGTGSGMGTL LISKVREEYP
hsTUBB1 (β1)      LGALFQPDSF VHGNSGAGNN WAKGHYTEGA ELIENVLEVV RHESESCDCL QGFQIVHSLG GGTGSGMGTL LMNKIREEYP
mmTUBB1 (β1)      LGVLFQPDSF VHGNSGAGNN WAKGHYTEGA ELIENVMDVV RRESESCDCL QGFQIVHSLG GGTGSGMGTL LMNKIREEYP
ggTUB6B (β1)      FGQIFRPDNF VFGQSGAGNN WAKGHYTEGA ELVDSVLDVV RKESESCDCL QGFQLTHSLG GGTGSGMGTL LISKIREEYP
hsTUBB2B (β2B)    FGQIFRPDNF VFGQSGAGNN WAKGHYTEGA ELVDSVLDVV RKESESCDCL QGFQLTHSLG GGTGSGMGTL LISKIREEYP
mmTUBB2B (β2B)    FGQIFRPDNF VFGQSGAGNN WAKGHYTEGA ELVDSVLDVV RKESESCDCL QGFQLTHSLG GGTGSGMGTL LISKIREEYP
ggTUBB1 (β2B)     FRQIFRPDNF VFGQSGAGNN WAKGHYTEGA ELVDSVLDVV RKEAESCDCL QGFQLTHSLG GGTGSGMGTL LISKIREEYP
hsTUBB4 (β4A)     FGQIFRPDNF VFGQSGAGNN WAKGHYTEGA ELVDAVLDVV RKEAESCDCL QGFQLTHSLG GGTGSGMGTL LISKIREEFP
scTUB2 (β)        IGNLFRPDNY IFGQSSAGNV WAKGHYTEGA ELVDSVMDVI RREAEGCDSL QGFQITHSLG GGTGSGMGTL LISKIREEFP

                                       180                   200                   220                   240
hsTUBB3 (β3)      DRIMNTFSVV PSPKVSDTVV EPYNATLSIH QLVENTDETY CIDNEALYDI CFRTLKLATP TYGDLNHLVS ATMSGVTTSL
mmTUBB3 (β3)      DRIMNTFSVV PSPKVSDTVV EPYNATLSIH QLVENTDETY CIDNEALYDI CFRTLKLATP TYGDLNHLVS ATMSGVTTSL
ggTUB4B (β3)      DRIMNTFSVV PSPKVSDTVV EPYNATLSIH QLVENTDETY CIDNEALYDI CFRTLKLATP TYGDLNHLVS ATMSGVTTSL
hsTUBB1 (β1)      DRIMNSFSVM PSPKVSDTVV EPYNAVLSIH QLIENADACF CIDNEALYDI CFRTLKLTTP TYGDLNHLVS LTMSGITTSL
mmTUBB1 (β1)      DRILNSFSVM PSPKVSDTVV EPYNAVLSIH QLIENTDACF CIDNEALYDI CFRTLRLTTP TYGDLNHLVS LTMSGITTSL
ggTUB6B (β1)      DRIMNTFSVM PSPKVSDTVV EPYNATVSVH QLVENTDETY CIDNEALYDI CFRTLKLTTP TYGDLNHLVS ATMSGVTTCL
hsTUBB2B (β2B)    DRIMNTFSVM PSPKVSDTVV EPYNATLSVH QLVENTDETY CIDNEALYDI CFRTLKLTTP TYGDLNHLVS ATMSGVTTCL
mmTUBB2B (β2B)    DRIMNTFSVM PSPKVSDTVV EPYNATLSVH QLVENTDETY CIDNEALYDI CFRTLKLTTP TYGDLNHLVS ATMSGVTTCL
ggTUBB1 (β2B)     DRIMNTFSVV PSPKVSDTVV EPYNATLSVH QLVENTDETY CIDNEALYDI CFRTLKLTTP TYGDLNHLVS ATMSGVTTCL
hsTUBB4 (β4A)     DRIMNTFSVV PSPKVSDTVV EPYNATLSVH QLVENTDETY CIDNEALYDI CFRTLKLTTP TYGDLNHLVS ATMSGVTTCL
scTUB2 (β)        DRMMATFSVL PSPKTSDTVV EPYNATLSVH QLVEHSDETF CIDNEALYDI CQRTLKLNQP SYGDLNNLVS SVMSGVTTSL

                                       260                   280                   300                   320
hsTUBB3 (β3)      RFPGQLNADL RKLAVNMVPF PRLHFFMPGF APLTARGSQQ YRALTVPELT QQMFDAKNMM AACDPRHGRY LTVATVFRGR
mmTUBB3 (β3)      RFPGQLNADL RKLAVNMVPF PRLHFFMPGF APLTARGSQQ YRALTVPELT QQMFDAKNMM AACDPRHGRY LTVATVFRGR
ggTUB4B (β3)      RFPGQLNADL RKLAVNMVPF PRLHFFMPGF APLTRRGSQQ YRALTVPELT QQMFDAKNMM AACDPRHGRY LTVATVFRGR
hsTUBB1 (β1)      RFPGQLNADL RKLAVNMVPF PRLHFFMPGF APLTAQGSQQ YRALSVAELT QQMFDARNTM AACDLRRGRY LTVACIFRGK
mmTUBB1 (β1)      RFPGQLNADL RKLAVNMVPF PRLHFFMPGF APLTAQGSQQ YRALSVAELT QQMFDARNIM AACDPRRGRY LTVACIFRGK
ggTUB6B (β1)      RFPGQLNADL RKLAVNMVPF PRLHFFMPGF APLTSRGSQQ YRALTVPELT QQMFDSKNMM AACDPRHGRY LTVAAIFRGR
hsTUBB2B (β2B)    RFPGQLNADL RKLAVNMVPF PRLHFFMPGF APLTSRGSQQ YRALTVPELT QQMFDSKNMM AACDPRHGRY LTVAAIFRGR
mmTUBB2B (β2B)    RFPGQLNADL RKLAVNMVPF PRLHFFMPGF APLTSRGSQQ YRALTVPELT QQMFDSKNMM AACDPRHGRY LTVAAIFRGR
ggTUBB1 (β2B)     RFPGQLNADL RKLAVNMVPF PRLHFFMPGF APLTSRGSQQ YRALTVPDLT QQMFDAKNMM AACEPGHGRY LTVAAVFRGR
hsTUBB4 (β4A)     RFPGQLNADL RKLAVNMVPF PRLHFFMPGF APLTSRGSQQ YRALTVPELT QQMFDAKNMM AACDPRHGRY LTVAAVFRGR
scTUB2 (β)        RYPGQLNSDL RKLAVNLVPF PRLHFFMVGY APLTAIGSQS FRSLTVPELT QQMFDAKNMM AAADPRNGRY LTVAAFFRGK

                                       340                   360                   380                   400
hsTUBB3 (β3)      MSMKEVDEQM LAIQSKNSSY FVEWIPNNVK VAVCDIPPRG LKMSSTFIGN STAIQELFKR ISEQFTAMFR RKAFLHWYTG
mmTUBB3 (β3)      MSMKEVDEQM LAIQSKNSSY FVEWIPNNVK VAVCDIPPRG LKMSSTFIGN STAIQELFKR ISEQFTAMFR RKAFLHWYTG
ggTUB4B (β3)      MSMKEVDEQM LAIQSKNSSY FVEWIPNNVK VAVCDIPPRG LKMSSTFIGN STAIQELFKR ISEQFTAMFR RKAFLHWYTG
hsTUBB1 (β1)      MSTKEVDQQL LSVQTRNSSC FVEWIPNNVK VAVCDIPPRG LSMAATFIGN NTAIQEIFNR VSEHFSAMFK RKAFVHWYTS
mmTUBB1 (β1)      MSTKEVDQQL LSIQTRNSNC FVEWIPNNVK VAVCDIPPRG LNMAATFLGN NTAIQELFTR VSEHFSAMFR RRAFVHWYTS
ggTUB6B (β1)      MSMKEVDEQM LNVQNKNSSY FVEWIPNNVK TAVCDIPPRG LKMSATFIGN STAIQELFKR ISEQFTAMFR RKAFLHWYTG
hsTUBB2B (β2B)    MSMKEVDEQM LNVQNKNSSY FVEWIPNNVK TAVCDIPPRG LKMSATFIGN STAIQELFKR ISEQFTAMFR RKAFLHWYTG
mmTUBB2B (β2B)    MSMKEVDEQM LNVQNKNSSY FVEWIPNNVK TAVCDIPPRG LKMSATFIGN STAIQELFKR ISEQFTAMFR RKAFLHWYTG
ggTUBB1 (β2B)     MSMKEVDEQM LNVQNKNSSY FVEWIPNNVK TAVCDIPPRG LKMSATFIGN STAIQELFKR ISEQFTAMFR RKAFLHWYTG
hsTUBB4 (β4A)     MSMKEVDEQM LSVQSKNSSY FVEWIPNNVK TAVCDIPPRG LKMAATFIGN STAIQELFKR ISEQFTAMFR RKAFLHWYTG
scTUB2 (β)        VSVKEVEDEM HKVQSKNSDY FVEWIPNNVQ TAVCSVAPQG LDMAATFIAN STSIQELFKR VGDQFSAMFK RKAFLHWYTS

                                       420                   440
hsTUBB3 (β3)      EGMDEMEFTE AESNMNDLVS EYQQYQD--A TAEEEGEMYE DDEEESEAQG PK-------
mmTUBB3 (β3)      EGMDEMEFTE AESNMNDLVS EYQQYQD--A TAEEEGEMYE DDDEESEAQG PK-------
ggTUB4B (β3)      EGMDEMEFTE AESNMNDLVS EYQQYQD--A TAEEEGEMYE DDEEESEQ-G AK-------
hsTUBB1 (β1)      EGMDINEFGE AENNIHDLVS EYQQFQDAKA VLEEDEEVTE EAEMEPEDKG H--------
mmTUBB1 (β1)      EGMDISEFGE AESDIHDLVS EYQQFQDVRA GLEDSEEDVE EAEVEAEDKD H--------
ggTUB6B (β1)      EGMDEMEFTE AESNMNDLVS EYQQYQD--A TADEQGEFEE EGEEDEA--- ---------
hsTUBB2B (β2B)    EGMDEMEFTE AESNMNDLVS EYQQYQD--A TADEQGEFEE EEGEDEA--- ---------
mmTUBB2B (β2B)    EGMDEMEFTE AESNMNDLVS EYQQYQD--A TADEQGEFEE EEGEDEA--- ---------
ggTUBB1 (β2B)     EGMDEMEFTE AESNMNDLVS EYQQYQD--A TAEEEGEFEE EAEEEAE--- ---------
hsTUBB4 (β4A)     EGMDEMEFTE AESNMNDLVS EYQQYQD--A TAEE-GEFEE EAEEEVA--- ---------
scTUB2 (β)        EGMDELEFSE AESNMNDLVS EYQQYQE--A TVEDDEEVDE NGDFGAPQNQ DEPITENFE
```

Supplementary Figure 1. Sequence comparison of β-tubulin isotypes in vertebrates and yeast. The sequences of β3 isotypes are highly conserved in vertebrates, showing only three amino acid residue differences between humans and chickens (coloured in yellow). In contrast, the sequence of human β3-tubulin differs from that of human β1, β2B, β4A-tubulin and yeast β-tubulin at 100, 34, 32 and 123 residues, respectively (coloured in pink). For each isotype, the sequence was obtained from UniProt (http://www.uniprot.org/): AAH03021 (human β3, hsTUBB3), AAH88749 (mouse β3, mmTUBB3), AAA49119 (chicken β3, ggTUB4B), AAH33679 (human β1, hsTUBB1), AAI47699 (mouse β1, mmTUBB1), AAA49124 (chicken β1, ggTUB6B), AAH63610 (human β2B, hsTUBB2B), BAE40722 (mouse β2B, mmTUBB2B), CAA23687 (chicken β2B, ggTUBB1), AAH13683 (human β4A, hsTUBB4), DAA12403 (yeast β, scTUB2). Amino acid sequences were aligned by clustalX.

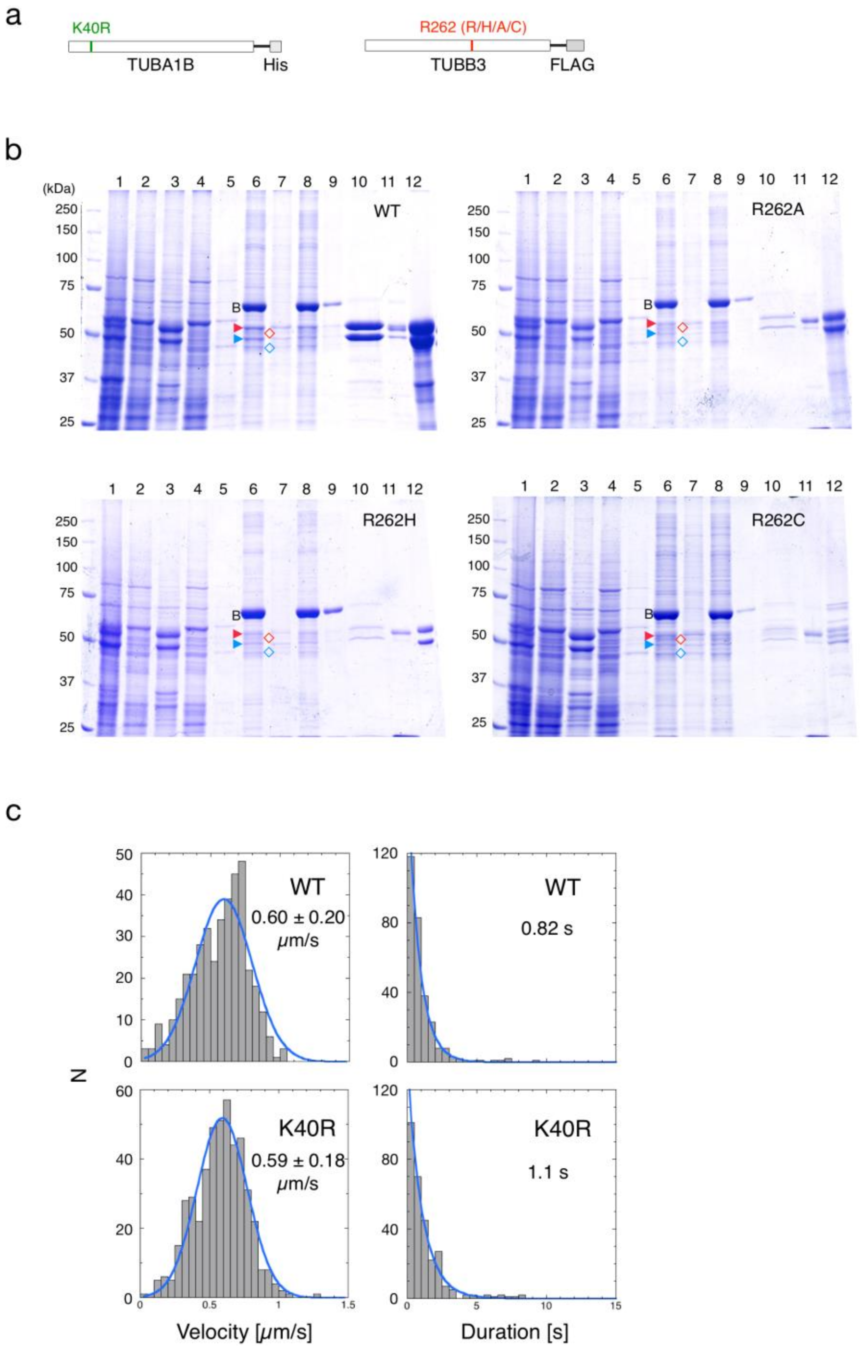

Supplementary Figure 2. Design and preparation of tubulin mutants. (**a**) Design of α- and β-tubulin constructs. These constructs are cloned into a single baculovirus vector. (**b**) SDS PAGE showing each preparation step of WT and R262A, R262H and R262C tubulin. Lanes: (1) Cell lysate, (2) soluble fraction and (3) precipitate after centrifugation, (4) His-affinity column flow-through, (5) His-affinity column wash, (6) His-affinity column eluent (supplemented with 2 mg ml^{-1} BSA), (7) the fraction that remained attached to the His-affinity column, (8) FLAG-affinity column flow-through, (9) FLAG-affinity column wash, (10) FLAG-affinity column eluent, (11) the fraction that remained attached to the FLAG-affinity column, and (12) after polymerization and centrifugation. ▶ and ▶, recombinant α- and β-tubulin; ◇ and ◇, endogenous α- and β-tubulin; B, BSA. (**c**) The effect of residue substitution at α-Lys40 residue on single-molecule motility of KIF5B. To prepare recombinant tubulin with a single post-translational modification state[1], the Lys40 of α-tubulin was substituted by Arg and treated as WT. The velocity and duration of α-K40R MTs was only slightly different from that of inherent WT, in which 10–30 % of α-tubulin is acetylated.

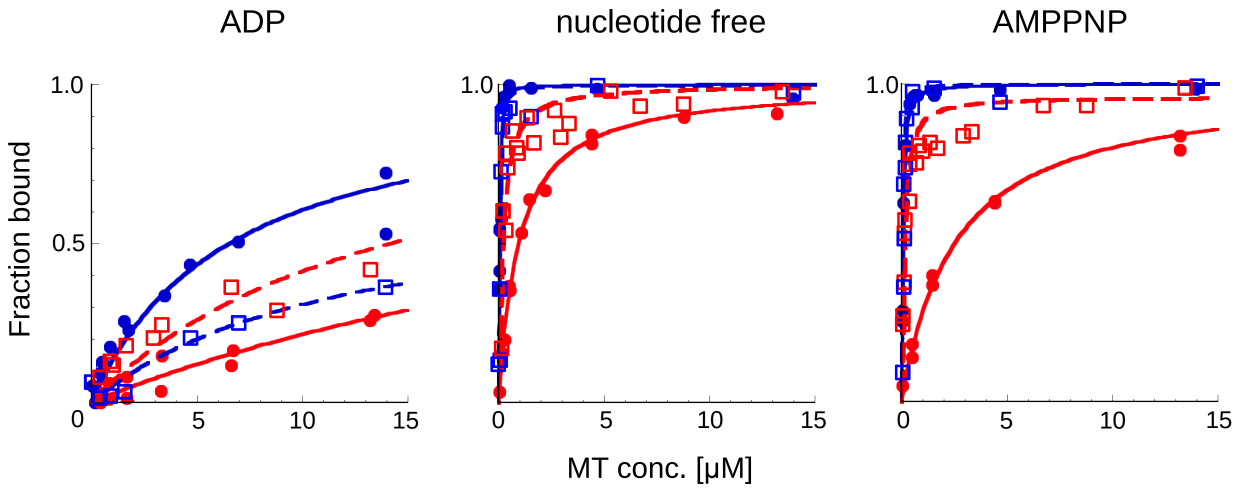

Supplementary Figure 3. Equilibrium dissociation constants of the KIF5B-MT complex. Binding of 0.1 μM single-headed HK349 KIF5B with varying concentrations of MTs. The fraction of HK349 in the MT pellet was plotted as a function of the total MT concentration. ●, WT KIF5B-WT MT; ●, WT KIF5B-R262A MT; □, D279R KIF5B-WT MT; □**,** D279R KIF5B-R262A MT. For each condition, measurements are repeated 2–3 times and all data from multiple rounds of measurements are plotted in the graph. The curves are the best fit to hyperbola with dissociation constant (K_d) values given in Supplementary Table 2 and Fig. 3f.

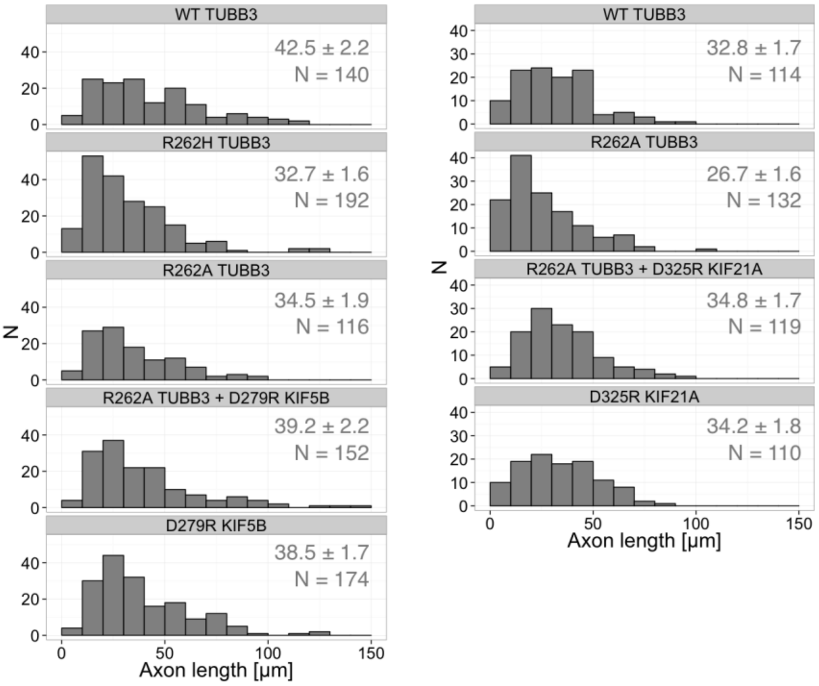

Supplementary Figure 4. Distribution of axon lengths when WT and mutant TUBB3, and/or mutant KIF5B/KIF21A were expressed in mouse cortex neurons. Data from two independent sets of experiment are shown. Numbers indicate mean ± s.e.m. lengths in μm.

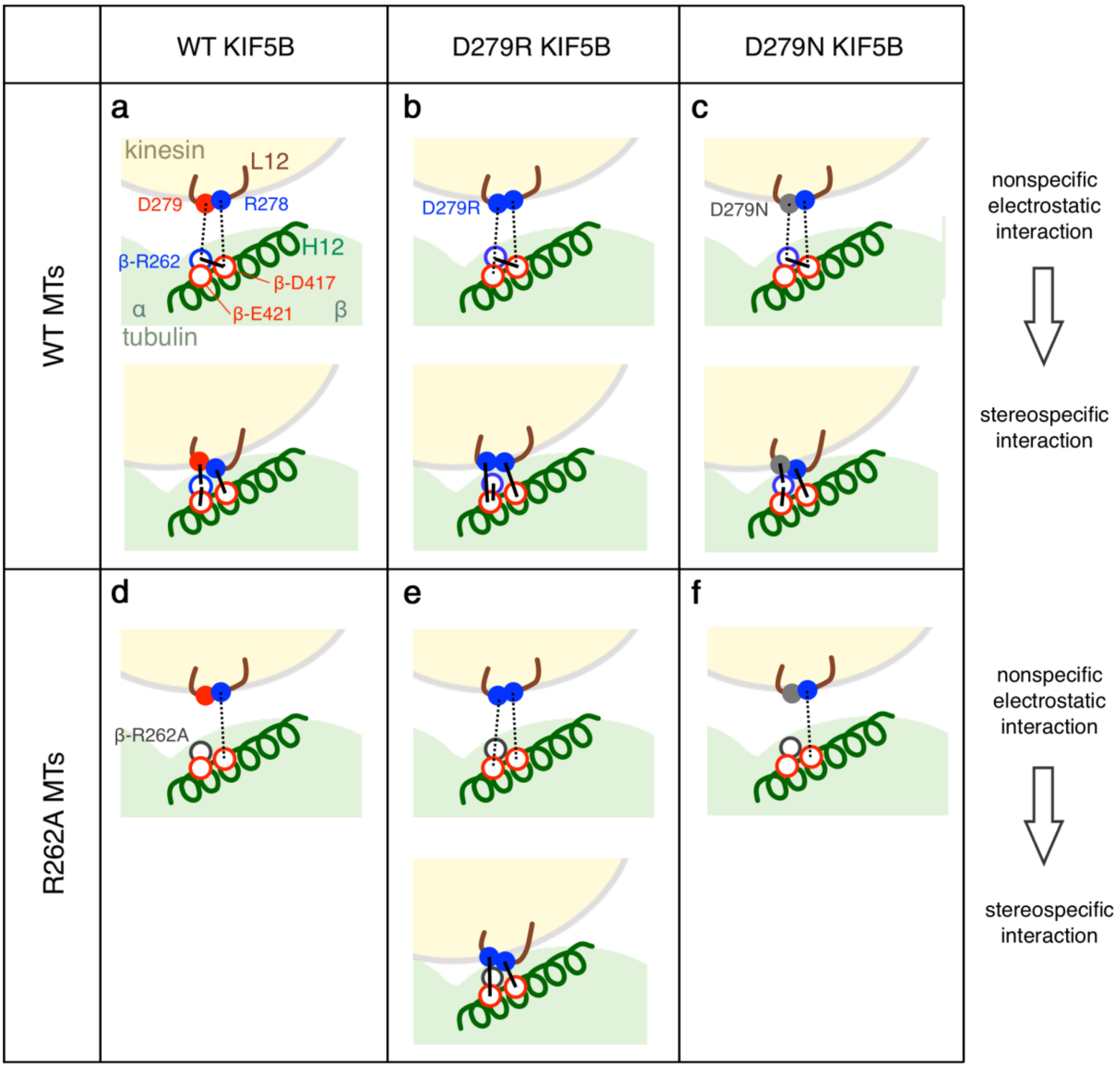

Supplemental Figure 5. Possible models for interactions between the L12 loop in KIF5B and the area near the residue β-R262 in TUBB3. Red, blue, and grey filled circles represent basic, acidic and neutral residues in KIF5B, respectively, whereas red, blue and grey unfilled circles represent basic, acidic and neutral residues in TUBB3, respectively. The dotted line indicates electrostatic attraction. Hydrogen bonds within tubulin molecule (indicated by thick lines) were predicted by using PyMOL, based on the simulated structure of αβ-tubulin dimer with or without β-R262A mutation (PDB: 1JFF)[2].

Both D279R and β-R262A mutations cause breaking of the salt bridge between D279 in KIF5B and R262 in TUBB3, which is formed in a WT-WT pair. However, the effect of each mutation on motility is different: While WT KIF5B was unable to move on β-R262A MT, D279R KIF5B was able to move on WT-MT (Fig. 3c, d, Table 1 in the main text). The result can be explained from the viewpoint of electrostatic interaction. In paired mutant WT KIF5B-β-R262A MT (d), it is difficult for residue D279 to find an alternative binding partner to replace β-R262A, because β-R262 is the only basic residue on an MT within an 8 Å distance from D279 (PDB: 4LNU). On the other hand, in paired mutant D279R KIF5B-WT MT (b), the D279R residue can easily find a binding partner, because the majority of kinesin-interacting tubulin residues are acidic. The local structure of the kinesin-MT interface[3] indicates that the repulsion between D279R and β-R262 could well be compensated for by the salt bridges D279R-β-E421 and R278-β-D417, allowing the pair (D279R KIF5B-WT MT) to stererospecifically bind.

Supplementary Table 1. Comparison of the parameters of microtubule-activated kinesin ATPase in charged-to-alanine tubulin and kinesin mutants

mutant	relative ATPase parameter		protein	reference
	k_{cat} (fold wt)	k_M(MT) (fold wt)		
WT-Tb	1.00 ± 0.03	1.00 ± 0.14	*Homo sapiens* TUBB3	This study[a]
β-Tb R262H	0.17 ± 0.03	9.71 ± 4.94		
β-Tb R262A	0.23 ± 0.01	10.3 ± 1.23		
WT-Tb	1.00 ± 0.03	1.00 ± 0.30	*S. cerevisiae* TUB2	Uchimura, et al, 2010[b]
α-Tb E414A	0.20 ± 0.02	0.78 ± 0.04		
α-Tb E415A	0.80 ± 0.09	2.39 ± 0.22		
α-Tb E417A	0.82 ± 0.03	1.00 ± 0.35		
α-Tb E420A	0.94 ± 0.03	1.83 ± 0.39		
β-Tb E410A	1.21 ± 0.11	6.39 ± 1.70		
β-Tb D417A	1.18 ± 0.05	5.96 ± 0.87		
kinesin R278A	0.66 ± 0.30	15.5 ± 5.45	*Homo sapiens* KIF5B	Woehlke, et al, 1997[c]
kinesin D279A	0.78 ± 0.14	0.44 ± 0.21		

[a] Values are mean ± errors of curve fitting

[b] Values are mean ± s.d. of 4–6 independent measurements[4]

[c] Values are mean ± s.d. of 2–5 independent measurements[5]

Supplementary Table 2. Equilibrium dissociation constants of the KIF5B-MT complex

microtubule	WT KIF5B			D279R KIF5B		
	ADP	nucleotide free	AMPPNP	ADP	nucleotide free	AMPPNP
WT	6.5 ± 0.7	$1.2 \pm 0.6 \times 10^{-2}$	$2.9 \pm 0.4 \times 10^{-2}$	$1.2 \pm 0.8 \times 10^{1}$	$1.2 \pm 0.6 \times 10^{-2}$	$1.0 \pm 0.4 \times 10^{-2}$
β-R262A	$3.6 \pm 0.3 \times 10^{1}$	$8.6 \pm 0.4 \times 10^{-1}$	2.5 ± 0.1	$1.4 \pm 0.2 \times 10^{1}$	$1.2 \pm 0.3 \times 10^{-1}$	$5.0 \pm 1.2 \times 10^{-2}$

Values are K_d in µM. Errors are those in curve fitting.

Supplementary Table 3. Summary of single-molecule motility assay of KIF21A on TUBB3 microtubules

microtubule	parameter	KIF21A	
		WT	D325R
WT	velocity [μm s^{-1}]	0.18 ± 0.32	0.24 ± 0.45
	duration [s]	7.70 ± 1.50	6.01 ± 1.74
	run length [μm]	1.56 ± 0.33	1.42 ± 0.41
β-R262H	velocity [μm s^{-1}]	UD	UD
	duration [s]	UD	UD
	run length [μm]	UD	UD
β-R262A	velocity [μm s^{-1}]	UD	0.16 ± 0.45
	duration [s]	UD	5.88 ± 0.89
	run length [μm]	UD	1.61 ± 0.73

Velocities, durations, and run lengths of the values in four independent experiments (N > 60 each, total N > 240; mean ± s.d.). UD, undetected. Motility was measured using BG-549 labeled dimeric construct of KIF21A (KIF21A-552). Only the population of KIF21A molecules showing directional movements was analyzed.

Supplementary References

1. Minoura, I., *et al.* Overexpression, purification, and functional analysis of recombinant human tubulin dimer. *FEBS Lett.* **587**, 3450-3455 (2013).
2. Fiser, A. & Sali, A. ModLoop: automated modeling of loops in protein structures. *Bioinformatics* **19**, 2500-2501 (2003).
3. Cao, L., Wang, W., Jiang, Q., Wang, C., Knossow, M. & Gigant, B. The structure of apo-kinesin bound to tubulin links the nucleotide cycle to movement. *Nat. Commun.* **5**, 5364 (2014).
4. Uchimura, S., Oguchi, Y., Hachikubo, Y., Ishiwata, S. & Muto, E. Key residues on microtubule responsible for activation of kinesin ATPase. *EMBO J.* **29**, 1167-1175 (2010).
5. Woehlke, G., Ruby, A. K., Hart, C. L., Ly, B., Hom-Booher, N. & Vale, R. D. Microtubule interaction site of the kinesin motor. *Cell* **90**, 207-216 (1997).
